# AHCYL1 Is Mediated by Estrogen-Induced ERK1/2 MAPK Cell Signaling and MicroRNA Regulation to Effect Functional Aspects of the Avian Oviduct

**DOI:** 10.1371/journal.pone.0049204

**Published:** 2012-11-07

**Authors:** Wooyoung Jeong, Jinyoung Kim, Suzie E. Ahn, Sang In Lee, Fuller W. Bazer, Jae Yong Han, Gwonhwa Song

**Affiliations:** 1 WCU Biomodulation Major, Department of Agricultural Biotechnology, Seoul National University, Seoul, Korea; 2 Center for Animal Biotechnology and Genomics and Department of Animal Science, Texas A&M University, College Station, Texas, United States of America; H. Lee Moffitt Cancer Center & Research Institute, United States of America

## Abstract

S-adenosylhomocysteine hydrolase-like protein 1 (AHCYL1), also known as IP_3_ receptor-binding protein released with IP_3_ (IRBIT), regulates IP_3_-induced Ca^2+^ release into the cytoplasm of cells. AHCYL1 is a critical regulator of early developmental stages in zebrafish, but little is known about the function of AHCYL1 or hormonal regulation of expression of the *AHCYL1* gene in avian species. Therefore, we investigated differential expression profiles of the *AHCYL1* gene in various adult organs and in oviducts from estrogen-treated chickens. Chicken *AHCYL1* encodes for a protein of 540 amino acids that is highly conserved and has considerable homology to mammalian AHCYL1 proteins (>94% identity). *AHCYL1* mRNA was expressed abundantly in various organs of chickens. Further, the synthetic estrogen agonist induced *AHCYL1* mRNA and protein predominantly in luminal and glandular epithelial cells of the chick oviduct. In addition, estrogen activated AHCYL1 through the ERK1/2 signal transduction cascade and that activated expression of AHCYL1 regulated genes affecting oviduct development in chicks as well as calcium release in epithelial cells of the oviduct. Also, microRNAs, *miR-124a, miR-1669, miR-1710* and *miR-1782* influenced *AHCYL1* expression *in vitro* via its 3′-UTR which suggests that post-transcriptional events are involved in the regulation of *AHCYL1* expression in the chick oviduct. In conclusion, these results indicate that *AHCYL1* is a novel estrogen-stimulated gene expressed in epithelial cells of the chicken oviduct that likely affects growth, development and calcium metabolism of the mature oviduct of hens via an estrogen-mediated ERK1/2 MAPK cell signaling pathway.

## Introduction

S-adenosylhomocysteine hydrolase-like protein 1 (AHCYL1) is a member of AHCY family of proteins involved in metabolism of S-adenosyl-L-homocysteine [1]. AHCYL1 consists of 540 amino acid residues and has a domain homologous to AHCY in the C-terminal region and multiple potential phosphorylation sites in the N-terminal region [2]. Unlike AHCY that catalyzes a reversible reaction for S-adenosylhomocysteine hydrolysis [3], [4], AHCYL1 does not have hydrolase activity for adenosylhomocysteine due to the substitution of important amino acids in the critical enzymatically active site [5], [6] although it has an AHCY-like domain in the C-terminal domain [1]. However, AHCYL1 plays important role in the inositol phospholipid (IP) signaling pathway by interacting with the inositol 1,4,5-trisphosphate (IP_3_) receptor, which is an intracellular Ca^2+^ release channel located on the endoplasmic reticulum [2], [5], [7], [8], [9], [10]. Therefore, AHCYL1 influences the IP_3_-induced Ca^2+^ signaling cascade essential for numerous cellular and physiological processes such as organ development, fertilization, and cell death [8], [11], [12]. Recently, Cooper and co-workers identified two zebrafish AHCYL1 orthologs and found that the function of AHCYL1 is different from AHCY and that it plays a key role in zebrafish embryogenesis in response to IP_3_ receptor function for release of intracellular calcium [1]. Although AHCYL1 is highly conserved among various species, little is known about its expression and functional roles in chickens or any other avian species.

In mammals, the oviduct undergoes diverse cellular and molecular changes in response to sex steroids during the estrous/menstrual cycle and peri-implantation period as these actions are pivotal to establishing an optimal microenvironment from gamete transport and embryonic development [13]. Of these steroid hormones, estrogen is the primary female sex hormone that controls a number of biological events including cell proliferation and differentiation, protection against apoptosis, and diabetes [14], [15]. To investigate mechanisms of action of sex steroids, their biological effects and signal transduction cascades, the chicken oviduct is an established model due to its responsiveness to steroid hormones [16]. The chicken oviduct is a highly differentiated linear organ with compartments that undergo structural, cellular and biochemical changes in response to sex hormones during egg formation and oviposition [17]. The oviduct of egg-laying hens consists of the infundibulum, magnum, isthmus, and shell gland essential for fertilization, production of egg-white proteins, formation of the soft shell membrane, and formation of the outer egg shell, respectively. In the chicken oviduct, estrogen induces both cell proliferation and differentiation, as well as anti-apoptotic effects on cells [18], [19]. In particular, estrogen stimulates formation of oviductal tubular glands and differentiation of epithelial cells into goblet and ciliated cells [20]. In addition, estrogen regulates the mechanism for Ca^2+^ release necessary for formation of the egg shell in the shell gland of the chicken oviduct [21], [22].

We used differential gene profiling data from the chicken oviduct to identify the avian homolog of the human *AHCYL1* transcript and found it to be highly expressed in chicks treated with the synthetic estrogen agonist diethylstilbestrol (DES) [23]. There is little known about the expression or function of AHCYL1 in most species, except for humans and zebrafish [1], [2], [6]. Therefore, the objectives of this study were to: 1) compare the primary sequences of chicken *AHCYL1* with those of selected mammalian species; 2) determine tissue- and cell-specific expression of *AHCYL1* gene in various organs of the mature chicken; 3) determine whether estrogen regulates expression of *AHCYL1* mRNA and protein during development of the chick oviduct; 4) determine whether AHCYL1 regulates calcium release and expression of genes related to development of the chicken oviduct through an estrogen-induced MAPK signaling pathway(s); and 5) investigate post-transcriptional regulation of AHCYL1 expression in the chicken oviduct. Results of this study provide novel insights into the *AHCYL1* gene with respect to its sequence, tissue specific expression and hormonal regulation of its expression during development of the chicken oviduct.

## Results

### Sequence Comparison, Pair-wise Alignment and Phylogenetic Tree Analysis of AHCYL1

The chicken *AHCYL1* gene spanned 10.3 kb on chromosome 26. The gene consists of 16 exons and the mRNA has 3,445 bp encoding a protein with 540 amino acid residues. The primary sequence of chicken *AHCYL1* was compared to those of six mammals and the zebrafish. Chicken AHCYL1 protein contained an NAD(P)-binding motif required for catalysis of S-adenosyl-L-homocysteine into adenosine and homocysteine as for mammalian AHCYL1s (Figure S1). This verified that AHCYL1 has a different function from AHCY because AHCYL1 lacks several binding sites for S-adenosyl-L-homocysteine, irrespective of the conserved cysteines required for a tight globular structure of AHCY and NAD^+^ binding motifs [1]. In pair-wise comparisons of chicken AHCYL1 proteins with seven other vertebrates, chicken AHCYL1 protein has high homology to mammalian AHCYL1 proteins (91–98%, Table 1). The phylogenetic tree was constructed using the neighbor-joining method (Figure S2). The human and orangutan *AHCYL1* genes clustered together and formed a larger cluster with mice and cattle, and an even larger cluster with sister groups was detected for dog and zebrafish. However, chicken AHCYL1 is in a separate branch, but closer to rat than to other mammalian species. These results indicate that chicken AHCYL1 diverged from mammalian AHCYL1 at very early stage in its evolution.

**Table 1:** Pairwise comparison of AHCYL1 proteins between chicken, mammalian and fish species.

Species	Symbol	Identity (%)
Chicken (*Gallus gallus*)	AHCYL1	–
vs. Human (*Homo sapiens*)	AHCYL1	96.3
vs. Orangutan (*Pongo abelii*)	AHCYL1	96
vs. Mouse (*Mus musculus*)	Ahcyl1	96.3
vs. Rat (*Rattus norvegicus*)	Ahcyl1	94
vs. Cattle (*Bos taurus*)	AHCYL1	97.8
vs. Dog (*Canis lupus familiaris*)	AHCYL1	98
vs. Zebrafish (*Danio rerio*)	ahcyl1	91

### *AHCYL1* mRNA Expression in Various Organs from Chickens

**Figure 1:**
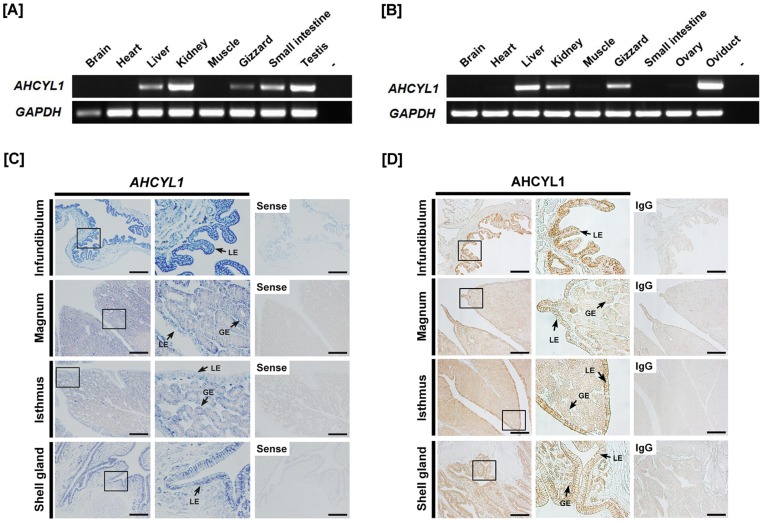
Expression of *AHCYL1* in chickens. [A and B] Expression of *AHCYL1* in various organs of male and female of chickens. Results of RT-PCR analysis using cDNA templates from different organs of male [A] and female [B] chickens with chicken *AHCYL1* and chicken *GAPDH*-specific primers. [C] *In situ* hybridization analyses of *AHCYL1* mRNAs in the chicken oviduct. Cross-sections of the four components of the chicken oviduct (infundibulum, magnum, isthmus and shell gland) were hybridized with antisense or sense chicken *AHCYL1* cRNA probes. [D] Immunoreactive AHCYL1 protein in the chicken oviduct. For the IgG control, normal mouse IgG was substituted for the primary antibody. Sections were not counterstained. Legend: LE, luminal epithelium; GE, glandular epithelium; *Scale bar* represents 100 µm. See *Materials and Methods* for complete description.

Tissue specific expression of *AHCYL1* mRNA in brain, heart, liver, kidney, muscle, gizzard, small intestine, ovary, oviduct and testis of 1- to 2- year-old males and females was determined by RT-PCR. High levels of expression of *AHCYL1* mRNA were detected in kidney and testis from male, and liver and oviduct from female chickens (Figures 1A and 1B), and lower expression in liver, gizzard and small intestine from males and kidney and gizzard from females. However, expression of *AHCYL1* mRNA was not detected in other organs analyzed regardless of sex. Based on differential gene profiling data of the chicken oviduct, the avian homolog of the human *AHCYL1* transcript is highly expressed in the oviduct of chicks treated with DES [23]. Since little is known about expression and function of AHCYL1 in the oviduct of any species [1], [2], [6], this study focused on its expression in the chicken oviduct.

### Localization of Chicken *AHCYL1* mRNA and Protein in Chicken Oviduct

*In situ* hybridization analysis was used to determine cell-specific localization of *AHCYL1* mRNA in the chicken oviduct. The oviduct of egg-laying hens includes the infundibulum (site of fertilization), magnum (production of components of egg-white), isthmus (formation of the shell membrane), and shell gland (formation of the egg shell). As illustrated in Figure 1C, *AHCYL1* mRNA was most abundant in the luminal epithelium (LE) of the infundibulum and shell gland, and it was also expressed at lower abundance in glandular epithelium (GE) and LE of the magnum and isthmus, and GE of the infundibulum and shell gland. Little or no mRNA was detected in stromal cells, blood vessels, immune cells or myometrium of the oviduct. Results of immunohistochemial analysis (Figure 1D) were consistent with results from *in situ* hybridization analyses. The AHCYL1 protein was abundant in LE of all segments of the oviduct, but less abundant in GE of magnum, isthmus and shell gland. The nonspecific mouse IgG, used as a negative control, did not detect AHCYL1 protein in any segment of the oviduct.

### Effects of DES on *AHCYL1* mRNA and Protein Expression in the Chicken Oviduct

**Figure 2:**
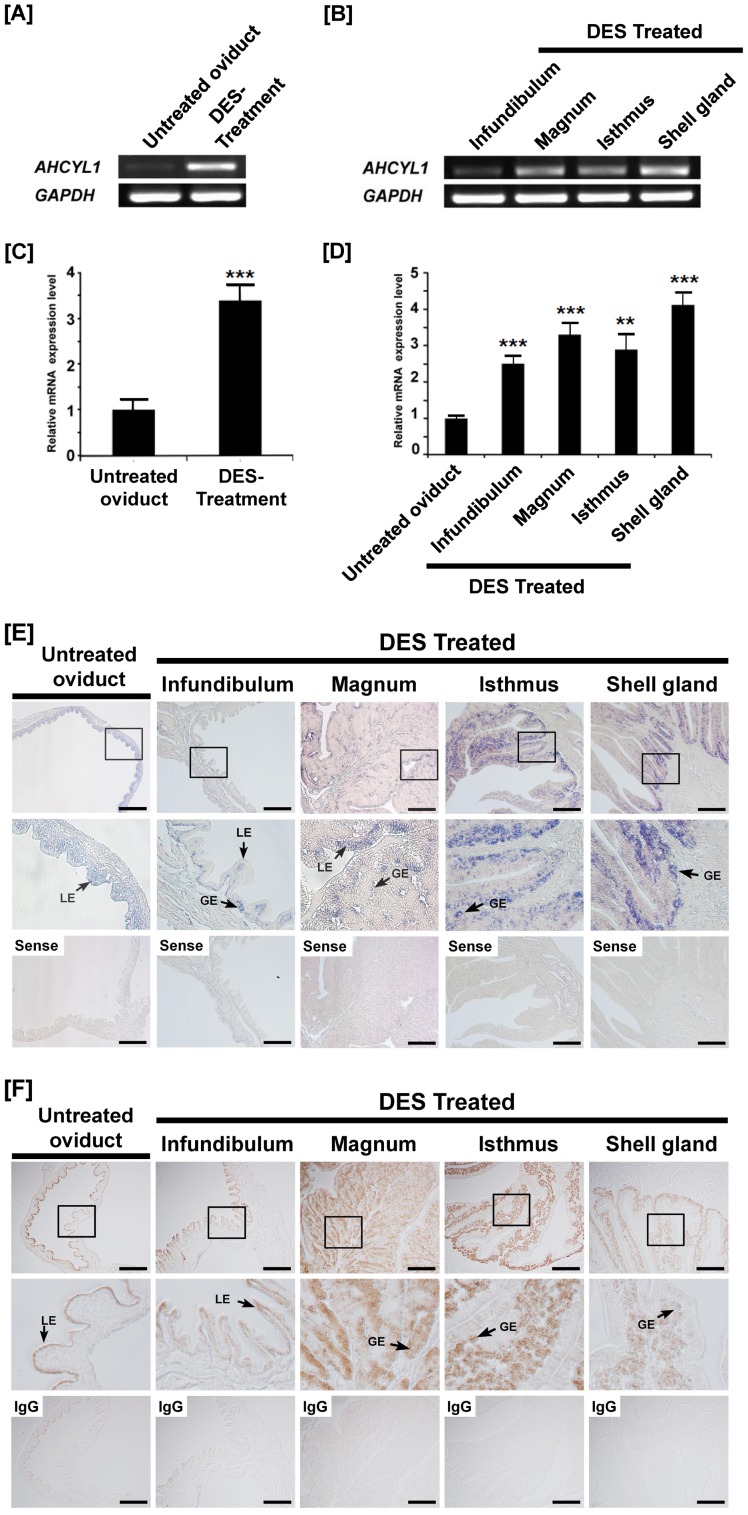
Effect of DES on tissue specific expression of chicken *AHCYL1*. Both RT-PCR [A and B] and q-PCR [C and D] analyses were performed using cDNA templates from DES-treated and untreated oviducts. These experiments were conducted in triplicate and normalized to control *GAPDH* expression. [E] *In situ* hybridization analyses revealed cell-specific expression of *AHCYL1* mRNA in oviducts of DES-treated and untreated chicks. Cross-sections of the four segments of chicken oviduct (infundibulum, magnum, isthmus, and shell gland) treated with DES or vehicle were hybridized with antisense or sense chicken *AHCYL1* cRNA probes. [F] Immunoreactive AHCYL1 protein in oviducts of DES-treated and untreated chicks. For the IgG control, normal goat IgG was substituted for the primary antibody. Sections were not counterstained. See *Materials and Methods* for complete description. Legend: Untreated oviduct, non-treated whole oviduct; DES Treatment, DES treated whole oviduct; LE, luminal epithelium; GE, glandular epithelium; *Scale bar* represents 100 µm. The asterisks denote statistically significant differences (***P<0.001 and **P<0.01).

Cell-specific expression of AHCYL1 in the oviductal segments of mature hens suggested regulation by estrogen during development of the chick oviduct. We reported that exogenous DES affects growth, development and differentiation of the chick oviduct and discovered candidate genes and pathways regulating oviduct development [35]. Therefore, we examined the effects of DES on *AHCYL1* expression in the chick oviduct. As shown in Figures 2A and 2B, semi-quantitative RT-PCR analysis indicated that DES increased *AHCYL1* mRNA in all segments of the 37-day-old chick oviduct. Further results from quantitative PCR revealed that DES induced a 3.3-fold increase (P<0.01) in oviductal *AHCYL1* mRNA as compared to 37-day-old chicks that were not treated with DES (Figure 2C). In addition, DES stimulated 2.3-, 3.4-, 2.6- and 4.3-fold increases (P<0.01) in *AHCYL1* mRNA in the infundibulum, magnum, isthmus, and shell gland, respectively (Figure 2D). *In situ* hybridization analyses revealed that *AHCYL1* mRNA is expressed specifically in superficial GE in close proximity to LE in all segments of the oviduct of chicks treated with DES (Figure 2E). *AHCYL1* mRNA is also expressed at lower abundance in LE of oviducts from untreated chicks. Consistent with results from *in situ* hybridization analyses, immunoreactive AHCYL1 protein was detected predominantly in superficial GE of magnum and isthmus, and to a lesser extent in GE of shell glands of oviducts treated with DES and LE of oviducts from untreated chicks (Figure 2F).

### DES Activates ERK1/2 Signal Transduction in Chicken Oviduct Cells

**Figure 3:**
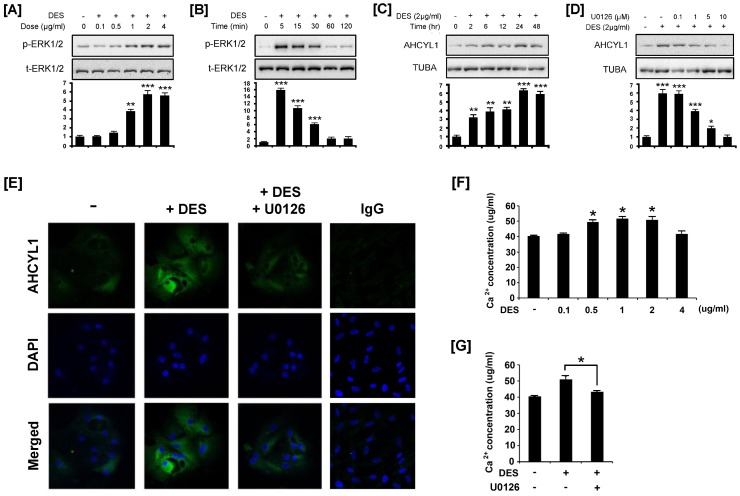
DES-induced phosphorylation of ERK1/2, stimulation of AHCYL1 and calcium release in chicken oviduct epithelial cells. [A and B] Dose-dependent and time-dependent expression of phosphorylated ERK1/2 in DES-treated chicken oviduct epithelial cells. Blots were imaged to calculate normalized values presented in graphs (bottom) by measurements of levels of phosphorylated protein relative to total protein. [C] In the DES-treated (2 µg/ml) and non-treated chicken oviduct cells, AHCYL1 protein levels were investigated to determine time-dependent effects of DES. [D] In chicken oviduct cells treated with DES (2 µg/ml) or both DES and an ERK1/2 inhibitor (U0126) for 24 h, according to results of a preliminary study to optimize time-dependent treatment effects, AHCYL1 protein decreased due to effects of U0126. In [C and D], blots were imaged to calculate the normalized values presented in graphs (bottom) for relative abundance of AHCYL1 protein and alpha-tubulin (TUBA) protein. [E] Immunofluorescence microscopy detected AHCYL1 protein in chicken oviduct epithelial cells treated with DES or both DES and an ERK1/2 inhibitor. AHCYL1 protein was barely detectable in untreated, as well as DES- and ERK1/2 inhibitor-treated cells, but abundant in cytoplasm of DES-treated oviduct epithelial cells. Cell nuclei were stained with DAPI (blue). All images were captured at 40X objective magnification. [F and G] Cells were grown in media with various concentration of DES for 24 h or both DES and an ERK1/2 inhibitor. Then, calcium concentration from the cells was measured. The asterisk denotes a significant effect (***P<0.001, **P<0.01 and *P<0.05). See *Materials and Methods* for complete description.

Epithelial cells from the chicken oviduct were isolated and cultured in the presence or absence of DES and subjected to protein extraction. Based on results from our preliminary experiments, we focused on MAPK signaling cascades, especially, the extracellular-signal regulated kinase1/2 (ERK1/2) signaling pathway. Based on dose–response experiments (Figure 3A), DES was used at 2 µg/ml in all experiments to determine cell signaling pathways mediating effects of DES on activation of ERK1/2 proteins. Western blot analyses of whole oviduct cell extracts with antibody to phosphorylated target proteins indicated that DES increased phospho-ERK1/2 (p-ERK1/2) 15.7-fold (P<0.01) over basal levels within 5 min and this effect was maintained to 30 min (Figure 3B). In addition, the same dose of DES increased AHCYL1 protein approximately 3-fold within 2 h and further stimulated it 6.3-fold over 24 h. Next, we examined effects of an ERK1/2 inhibitor (U0126) on the ability of DES to increase synthesis of AHCYL1 protein (Figure 3C). As illustrated in Figure 3D, 1 to 10 µM U0126 decreased the abundance of AHCYL1 protein in response to DES treatment. To verify these results, we performed immunofluorescence analyses and compared expression patterns of AHCYL1 protein in chick oviduct cells cultured in the presence or absence of DES or presence of DES with U0126 (Figure 3E). Immunoreactive AHCYL1 protein was most abundant in the cytoplasm of chicken oviduct epithelial cells treated with DES, but detectable at lower abundance in the cytoplasm of cells receiving no treatment or DES treatment with U0126. Furthermore, DES stimulated calcium release from epithelia cell of the magnum of the chicken oviduct in a dose-dependent manner (Figure 3F). However, calcium release was reduced in these cells when cultured in the presence of both DES and U0126 as compared to DES alone (Figure 3G).

### AHCYL1 Knockdown and Expression of Genes Related to Oviduct Development in Response to Estrogen

**Figure 4:**
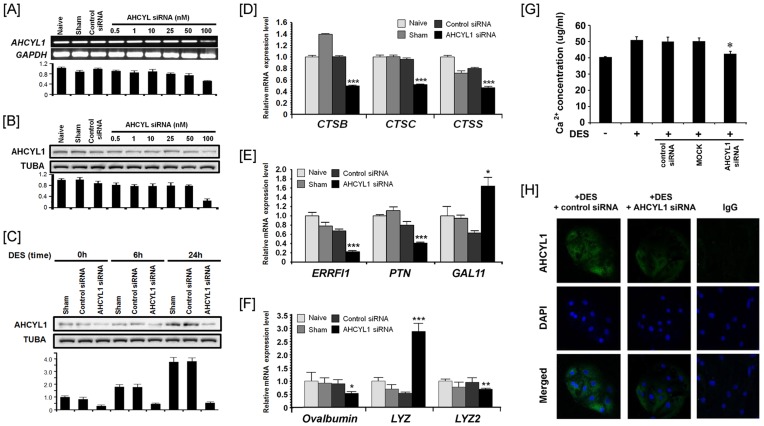
AHCYL1 knockdown decreased expression of genes associated with oviduct development and production of egg white proteins. [A] In the control group (naïve, sham and control siRNA treatment) and AHCYL1 silencing group (dose-dependent manner), *AHCYL1* mRNA levels were quantified by RT-PCR and quantitative RT-PCR analyses. [B] In the control group (naïve, sham and control siRNA treatment) and AHCYL1 silencing group (dose-dependent), immunoreactive AHCYL1 protein was quantified by western blotting. [C] The effects of DES treatment (time-dependent manner) on control cells and cells in which AHCYL1 was silenced is shown in Panels D to F. Total RNA isolated from chicken oviduct epithelial cells treated with AHCYL1 siRNA (100nM) affected expression of *CTSB, CTSC, CTSS, ERRFI1*, *PTN*, *GAL11, ovalbumin, LYZ* and *LYZ2* mRNAs as determined using quantitative RT-PCR analyses. Legend: CTSB, cathepsin B; CTSC, cathepsin C; CTSS, cathepsin S; ERRFI1, ERBB receptor feedback inhibitor 1; PTN, pleiotrophin, GAL11; gallinacin 11, LYZ; lysozyme. [G] Cells were grown in medium with the absence and presence of DES with siRNAs and then changes in amount of calcium released from the cells was measured. [H] Immunofluorescence microscopy detected AHCYL1 protein in chicken oviduct epithelial cells treated with DES with siRNAs. Cell nuclei were stained with DAPI (blue). All images were captured at 40X objective magnification. The asterisks denote statistically significant differences (***P<0.001, **P<0.01 and *P<0.05). See *Materials and Methods* for complete description.

The constitutive expression of AHCYL1 after transfection was not significantly different in chicken oviduct epithelial cells at 0.5, 1, 10, 25 and 50 nM of AHCYL1-specific siRNA. However, AHCYL1 protein expression was inhibited 25.3% at 48 h post-transfection with AHCYL1 siRNA at 100 nM (Figures 4A and 4B). Therefore, we investigated whether DES affects levels of AHCYL1 expression in chick oviduct cells transfected with AHCYL1 siRNA for 48 h and then treated with 2 ug/ml DES for 0, 6 or 24 h. Cell transfected with AHCYL1-specific siRNA had less AHCYL1 compared to sham-treated cells (p<0.001) and cells transfected with control siRNA (p<0.001) at each time point (Figure 4C). Sham and control siRNA cells treated with DES for 6 and 24 h had a greater increase in AHCYL1 protein compared to sham and control siRNA cells not treated (0 h) with DES (p<0.05). To examine expression of genes related to chicken oviduct development and major egg white proteins in response to estrogen, we examined expression of *cathepsin B* (*CTSB*), *CTSC, CTSS, ERBB receptor feedback inhibitor 1* (*ERRFI1*), *pleiotrophin* (*PTN*), *gallinacin 11* (*GAL11*), *ovalbumin, lysozyme* (*LYZ*) and *LYZ2* using quantitative PCR analyses. As illustrated in Figure 4D to 4F, the expression levels for *CTSB, CTSC, CTSS, ERRFI1*, *PTN*, *ovalbumin* and *LYZ2* mRNAs were decreased significantly by AHCYL1 knockdown as compared to naïve, sham and control siRNA treatments: *CTSB* to 0.49- (p<0.001), *CTSC* to 0.51- (p<0.001), *CTSS* to 0.46- (p<0.001), *ERRFI1* to 0.22- (p<0.001), *PTN* to 0.41- (p<0.001), *ovalbumin* to 0.61- (p<0.05) and *LYZ2* to 0.31-fold (p<0.01). However, the expression of *GAL11* and *LYZ* mRNAs increased significantly in response to AHCYL1 knockdown: *GAL11* to 1.64- (p<0.05) and *LYZ* to 2.88-fold (p<0.001). In addition, calcium release was reduced significantly in response to AHCYLI knockdown as compared to control or control siRNA treated cells (Figure 4G). Moreover, immunoreactive AHCYL1 protein was less abundant in the cytoplasm of the chicken oviduct cells receiving DES treatment and AHCYL1 siRNA transfection as compared to treatment with DES and the control siRNA (Figure 4H).

### Post-transcriptional Action of miRNAs on AHCYL1

**Figure 5:**
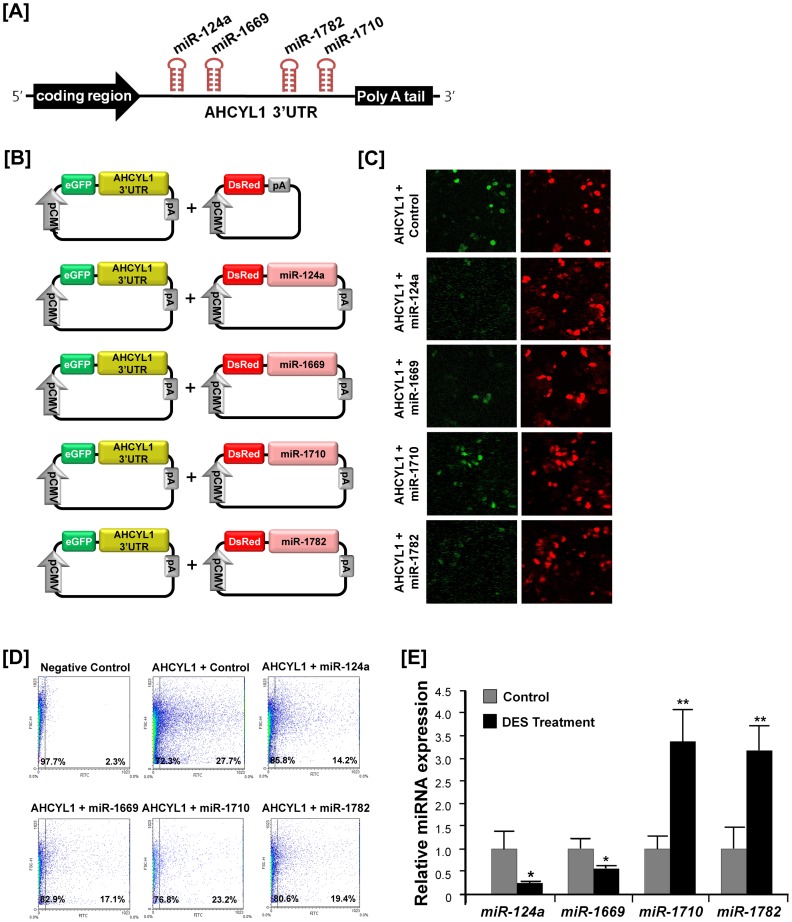
*In vitro* target assay of *miR-124a, miR-1602, miR-1612, miR-1669, miR-1710* and *miR-1782 on AHCYL1* transcript. [A] Diagram of *miR-124a, miR-1669, miR-1710 and miR-1782* binding sites in *AHCYL1* 3′-UTR. [B] Expression vector maps for eGFP with *AHCYL1* 3′UTR and Ds-Red with each miRNA. The 3′-UTR of the *AHCYL1* transcript was subcloned between the eGFP gene and the polyA tail to generate the fusion construct of the GFP transcript following the miRNA target 3′-UTR (pcDNA-eGFP-3′UTR) (upper panel) and miRNA expression vector was designed to co-express DsRed and each miRNA (pcDNA-DsRed-miRNA) (lower panel). [C and D] After co-transfection of pcDNA-eGFP-3′UTR for the *AHCYL1* transcript and pcDNA-DsRed-miRNA for the *miR-124a, miR-1669, miR-1710 and miR-1782*, the fluorescence signals of GFP and DsRed were detected using fluorescent microscopy [C] and FACS [D]. [E] q-PCR analyses were performed using cDNA templates from DES-treated and untreated chicken oviducts (mean ± SEM). These experiments were conducted in triplicate and normalized to control U6 snRNA expression. See *Materials and Methods* for complete description. The asterisks denote statistically significant differences (**P<0.01 and *P<0.05).

Expression of *AHCYL1* may be regulated at the post-transcriptional level by microRNAs (miRNAs); therefore, we performed a miRNA target validation assay. Analysis of potential miRNA binding sites within the 3′-UTR of the *AHCYL1* gene using the miRNA target prediction database (miRDB; http://mirdb.org/miRDB/) revealed six putative binding sites for *miR-124a, miR-1602, miR-1612, miR-1669, miR-1710* and *miR-1782* (Figure 5A). Therefore, we determined whether these miRNAs influenced *AHCYL1* expression via its 3′-UTR. A fragment of the *AHCYL1* 3′-UTR harboring binding sites for the miRNAs were cloned in downstream of the green fluorescent protein (GFP) reading frame, thereby creating a fluorescent reporter for function of the 3′-UTR region (Figure 5B). After co-transfection of eGFP-*AHCYL1* 3′-UTR and DsRed-miRNA, the intensity of GFP expression and percentage of GFP-expressing cells were analyzed by fluorescence microscopy and FACS. As illustrated in Figures 5C and 5D, in the presence of *miR-124a, miR-1669, miR-1710, miR-1782*, the intensity and percentage of GFP-expressing cells (27.7% in control vs. 14.2% in *miR-124a*, 17.1% in *miR-1669*, 19.4% in the *miR-1782* and 23.2% in *miR-1710*) were decreased. However, in the presence of *miR-1602* and *miR-1612*, there was no significant decrease in green fluorescence compared to the control (data not shown). Further results from quantitative PCR revealed that DES induced a 3.38- and 3.18-fold increase (P<0.01) in expression of *miR-1710* and *-1782*, respectively, in the oviduct as compared to control chicks (Figure 5E). However, expression of *miR-124a* and *miR-1669* was decreased in DES-treated oviducts (P<0.05). These results indicate that *miR-124a, miR-1669, miR-1710* and *miR-1782*, influence *AHCYL1* expression *in vitro* via its 3′-UTR which suggests that post-transcriptional events regulate or influence *AHCYL1* expression in the chick oviduct.

## Discussion

Results of the present study are novel as they provide the first comparisons among chicken and mammalian *AHCYL1* genes with respect to structure, phylogenetic evolution, tissue specific expression of *AHCYL1* mRNA and protein, and regulation of expression by estrogen in an ERK1/2-dependent cell signaling cascade. Our results also indicate that AHCYL1 is post-transcriptionally regulated by several miRNAs and knockdown of AHCYL1 results in down-regulation of genes critical to development of the chick oviduct in response to estrogen in chicks. These results support our hypothesis that AHCYL1 is required for growth, development and functional aspects of the mature oviduct of hens in response to estrogen during their reproductive cycle.

We reported that differential gene profiling data of the chick oviduct showed that the avian homolog of human *S-adeonosylhomocysteine hydrolase like protein 1* (*AHCYL1*) transcript is highly expressed in chicks treated with DES [35]. AHCYL1 regulates numerous important cellular processes, especially Ca^2+^-dependent processes, by modulating concentrations of Ca^2+^ in cytoplasm of cells [11], [12]. However, little is known about expression and function of AHCYL1 in the oviduct of any species [1], [2], [6] although AHCYL1 has potential role(s) in many important biological events such as development, fertilization, gene expression, secretion and cell death [8], [11], [12].

In the present study, we found that the chicken AHCYL1 gene consists of 16 exons encoding 540 amino acid residues and that it has high homology (greater than 90%) to mammalian AHCYL1 proteins (Table 1). In addition, expression of *AHCYL1* mRNA in kidney, liver, testis, oviduct, and to a lesser extent, in gizzard and small intestine of chickens was found. However, expression was not detected in other organs analyzed in either sex. Furthermore, as illustrated in Figures 1C and 1D, AHCYL1 mRNA and protein were most abundant in the LE of the infundibulum and shell gland, and at lower abundance in GE and LE of the magnum and isthmus, and GE of the infundibulum and shell gland of the oviduct. These results indicate that cell- and tissue-specific expression of *AHCYL1* may be associated with functional mechanism(s) of chicken oviduct functions including calcium metabolism for formation of the egg shell and oviposition (egg laying).

Generally, the biological actions of estrogen are mediated by its cognate nuclear receptors, estrogen receptors alpha (ESR1) and beta (ESR2) which activate and recruit a variety of transcription factors with estrogen response elements to the 5′ upstream region of target genes [15], [16]. Indeed, several steroid hormones, including estrogen, are involved in many physiological and developmental events requiring modification of cell-type and tissue-specific gene expression [16], [36]. Although various animal models have been used to investigate developmental and hormonal mechanism of oviduct growth, development and differentiation, the most well studied and informative model is the chick oviduct [16]. During development of the chicken oviduct, estrogen stimulates proliferation and cytodifferentiation of epithelial cells to tubular gland cells and expression of oviduct-specific genes [37], [38]. In particular, the differentiated tubular gland cells of the magnum synthesize and secrete the egg-white proteins including ovalbumin, lysozyme, ovotransferrin, ovomucoid and avidin during egg formation [39]. Indeed, the magnum is the most estrogen-responsive segment of the chicken oviduct. The administration of exogenous estrogen to neonatal chicks stimulates an 8-fold increase in wet weight of the magnum within three days [40]. Consistent with these results, we reported that exogenous DES affects growth, development and differentiation of the chicken oviduct [26] and discovered candidate genes and pathways regulating oviduct development in chickens [35]. In the present study, as illustrated in Figure 2, DES treatment increased *AHCYL1* mRNA and protein in LE of the infundibulum and magnum, and GE of the isthmus and shell gland of the chick oviduct. These results strongly support our hypothesis that estrogen-mediated *AHCYL1* gene expression plays a crucial role(s) in growth, differentiation and function of the chicken oviduct.

Results of the current study revealed that estrogen stimulates activation of ERK1/2 phosphorylation, expression of AHCYL1, and calcium release by oviduct cells of chicks. Mitogen-activated protein kinases (MAPKs) are highly conserved in most organisms and respond to various extracellular stimuli such as mitogens, heat shock, stress and cytokines [41]. Among the three well-characterized subfamilies of MAPKs, the ERK1/2 MAPK pathway plays important roles in growth and differentiation processes of female reproductive organs during early pregnancy, including embryonic and placental development [42], [43], [44], [45]. However, little is known about the ERK1/2 MAPK signal cascade in growth, development and differentiation of female reproductive tract such as oviduct and uterus. In the present study, DES induced a rapid increase in phosphorylation of ERK1/2 MAPK by 5 min and this effect was maintained to 30 min before declining by 60 min (Figure 3). In addition, the same dose of DES increased AHCYL1 protein 3-fold within 2 h and 6.3-fold by 24 h and induced calcium release in a dose-dependent manner. Meanwhile, treatment of chicken oviduct epithelial cells with both an ERK1/2 inhibitor (U0126) and DES decreased AHCYL1 protein in the cytoplasm of those cells and inhibited calcium release despite DES treatment. These results strongly suggest that estrogen influences development and differentiation of the chick oviduct by activating AHCYL1 and calcium release in an ERK1/2 MAPK-dependent manner.

RNA interference methods such as the siRNA-mediated recognition of homologous target mRNA molecule have been used successfully in biological research to examine effects of silencing target genes [46]. In this study, we determined that AHCYL1 knockdown decreases expression of several genes associated with oviduct development and differentiation including several members of the cathepsin (CTS) family of lysosomal proteases. CTSs degrade extracellular matrix (ECM) molecules including collagens, laminin, fibronectin and proteoglycans and they are also involved in catabolism of intracellular proteins and processing of pro-hormones. In addition, the CTSs regulate intracellular protein metabolism [47], bone resorption [48] and antigen presentation [49], as well as cell transformation, differentiation, motility, and adhesion [50]. In the present study, the expression of *cathepin B* (*CTSB), CTSC,* and *CTSS* mRNAs was significantly decreased by AHCYL1 knockdown compared to naïve, sham and control siRNA treatments (Figure 4D). Furthermore, the expression of *ERBB receptor feedback inhibitor 1 (ERRFI1), pleiotrophin (PTN)*, *gallinacin 11 (GAL11), ovalbumin, lysozyme (LYZ)* and *LYZ2* mRNAs, which are estrogen-induced genes or genes for egg white proteins expressed in the oviduct epithelial cells of the chicken were also significantly affected by AHCYL1 knockdown. These results suggest that estrogen-induced AHCYL1 regulates downstream genes for oviduct growth/remodeling and maintenance of oviduct function during the reproductive cycle of chickens.

MicroRNAs (miRNAs), as post-transcriptional regulators, play essential roles in a wide variety of biological processes including vertebrate growth, development and differentiation [51]. In the current study, we performed a miRNA target validation assay based on the hypothesis that *AHCYL1* expression is regulated at the post-transcriptional level by miRNAs. As illustrated in Figure 5, co-transfection of eGFP-*AHCYL1* 3′-UTR and DsRed-miRNA decreased the percentage of GFP-positive cells and GFP fluorescence density in *miR-124a, miR-1669, miR-1710* and *miR-1782* transfected cells, but not in cell transfected with *miR-1602* and *miR-1612* when compared to untreated control cells. However, as illustrated in Figure 5E, the *in vivo* DES-mediated decrease in *miR-124a* and *miR-1669* supports this hypothesis, whereas the DES-mediated increase in *miR-1710* and *miR-1782* is inconsistent with the *in vitro* data. These results indicate that *miR-1710* and *miR-1782* may act indirectly or regulate expression of other DES-regulated genes in vivo. Collectively, these four miRNAs_influence *AHCYL1* expression *in vitro* via its 3′-UTR which suggests that post-transcriptional regulation influences *AHCYL1* expression in the chick oviduct. In addition, we propose that, of these four miRNAs, *miR-124a* and miR-1669 are closely related to the regulatory pathways of oviduct development and differentiation in chickens; however, this requires further investigation.

**Figure 6:**
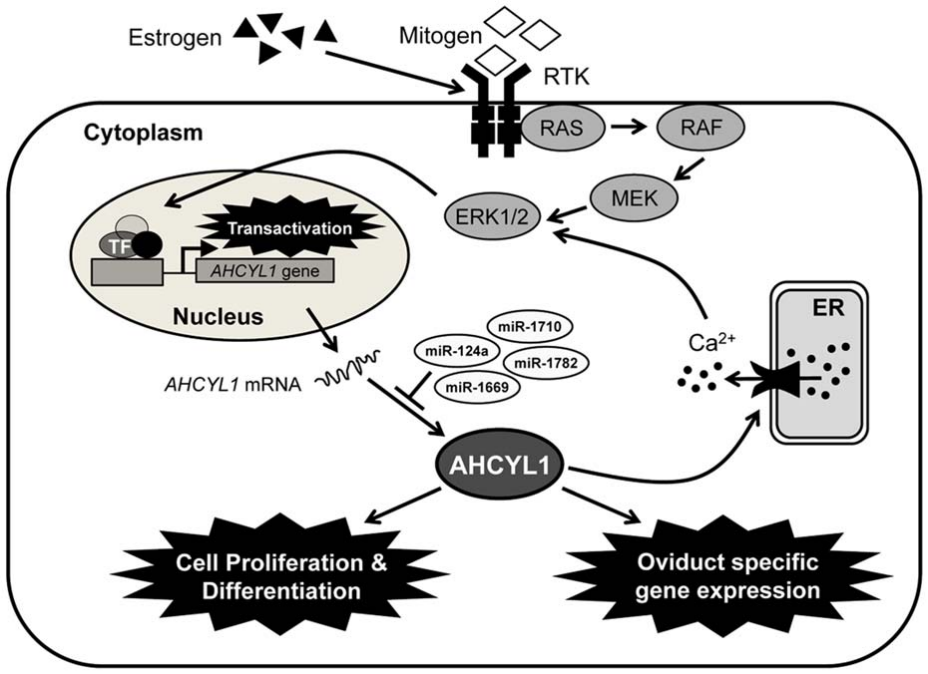
Schematic illustrating the current working hypothesis on estrogen-induced ERK1/2 MAPK signaling cascades in chicken oviduct cells. Evidence from the present study indicates that estrogen stimulates the classical estrogen- and alternative ERK1/2 MAPK signaling pathways. Legend: RTK, receptor tyrosine kinase; RAS, synaptic Ras-GTPase-activating protein; RAF (also known as MAPK3), mitogen-activated protein kinase (MAPK) kinase kinase; MEK (also known as MAPK2), MAPK kinase; ERK1/2, extracellular signal-regulated kinase; ERE, estrogen response element; ER, endoplasmic reticulum.

Based on the collective results from the present studies, we propose a model (Figure 6) in which estrogen activates receptor tyrosine kinase (RTK) and phosphorylated RTK activates RAS-RAF-MEK to stimulate the ERK1/2 signal transduction cascade to effect expression of genes affecting growth- and/or development of the chick oviduct and to stimulate oviduct-specific genes for the production of egg white proteins and calcium release during egg formation. In conclusion, results of the present study provide important insights into the mechanism by which AHCYL1 regulates growth, development and functional aspects of the mature oviduct of hens in response to estrogen-mediated ERK1/2 MAPK cell signaling during their reproductive cycle.

## Materials and Methods

### Experimental Animals and Animal Care

The experimental use of chickens for this study was approved by the Institute of Laboratory Animal Resources, Seoul National University (SNU-070823-5). White Leghorn (WL) hens, roosters, and chicks were subjected to standard management practices at the University Animal Farm, Seoul National University, Korea. All chickens were exposed to a light regimen of 15 h light and 9 h dark with *ad libitum* access to feed and water.

### Tissue Samples

Following euthanasia of the WL hens and roosters, tissue samples were collected from brain, heart, liver, kidney, muscle, small intestine, gizzard, ovary, oviduct and testis of 1- to 2- year-old males (n = 5) and females (n = 5). The collected samples were frozen or fixed in 4% paraformaldehyde for further analyses. Frozen tissue samples were cut into 5- to 7- mm pieces, frozen in liquid nitrogen vapor, and stored at −80°C. The other samples were cut into 10 mm pieces and fixed in fresh 4% paraformaldehyde in PBS (pH 7.4). After 24 h, fixed tissues were changed to 70% ethanol for 24 h and then dehydrated and embedded in Paraplast-Plus (Leica Microsystems, Wetzlar, Germany). Paraffin-embedded tissues were sectioned at 5 µm.

### Diethylstilbestrol (DES) Treatment and Oviduct Retrieval

Female chicks were identified by PCR analysis using W chromosome-specific primer sets [24]. Treatment with DES and recovery of the oviduct were performed as reported previously [25], [26]. Briefly, a 15 mg DES pellet was implanted subcutaneously in the abdominal region of 1-week-old female chicks for 10 days. The DES pellet was removed for 10 days, and a 30 mg dose was administered for 10 additional days. Five 37-day-old chicks in each group were euthanized using 60%–70% carbon dioxide. Subsets of these samples were frozen or fixed in 4% paraformaldehyde for further analyses. Paraffin-embedded tissues were sectioned at 5 µm.RNA Isolation.

Total cellular RNA was isolated from frozen tissues using Trizol reagent (Invitrogen, Carlsbad, CA) according to the manufacturer’s recommendations. The quantity and quality of total RNA was determined by spectrometry and denaturing agarose gel electrophoresis, respectively.

### Sequence Analysis

For pair-wise comparisons and multiple sequence alignment, the amino acid sequences encoded by *AHCYL1* genes from each species were aligned using Geneious Pro Version 5.04 [27] with default penalties for gap and the protein weight matrix of BLOSUM (Blocks Substitution Matrix). A phylogenetic tree was constructed using the neighbor-joining method [28] of the Geneious Pro Version 5.04 [27]. To determine the confidence level for each internal node on the phylogenetic tree, 1,000 nonparametric bootstrap replications were used [29].

### Semiquantitative RT-PCR Analysis

The expression level of *AHCYL1* mRNA in various organs from chickens, including the oviduct, was assessed using semi-quantitative RT-PCR as described previously [30]. The cDNA was synthesized from total cellular RNA (2 ug) using random hexamer (Invitrogen, Carlsbad, CA) and oligo (dT) primers and AccuPower® RT PreMix (Bioneer, Daejeon, Korea). The cDNA was diluted (1∶10) in sterile water before use in PCR. For *AHCYL1*, the sense primer (5′-TTT GGA GGG AAG CAA GTG GC-3′) and antisense primer (5′-GCT CAA TCA GAG CCA GAG CC-3′) amplified a 481-bp product. For *GAPDH* (housekeeping gene; glyceraldehyde 3-phosphate dehydrogenase), the sense primer (5′-TGC CAA CCC CCA ATG TCT CTG TTG-3′) and antisense primer (5′-TCC TTG GAT GCC ATG TGG ACC AT-3′) amplified a 301-bp product. The primers, PCR amplification and verification of their sequences were conducted as described previously [30]. PCR amplification was conducted using approximately 60 ng cDNA as follows: 1) 95°C for 3 min; 2) 95°C for 20 sec, 60°C for 40 sec, and 72°C for 1 min for 35 cycles (*AHCYL1*) and 30 cycles (*GAPDH*); and 3) 72°C for 5 min. The amount of DNA present was quantified by measuring the intensity of light emitted from correctly sized bands under UV light using a Gel Doc™ XR+ system with Image Lab™ software (Bio-Rad).

### Quantitative PCR Analysis

Total RNA was extracted from each oviduct of untreated and DES-treated chicks using TRIzol (Invitrogen) and purified using an RNeasy Mini Kit (Qiagen). Complementary DNA was synthesized using AccuPower® RT PreMix (Bioneer, Daejeon, Korea). Gene expression levels were measured using SYBR® Green (Sigma, St. Louis, MO, USA) and a StepOnePlus™ Real-Time PCR System (Applied Biosystems, Foster City, CA, USA). The PCR conditions were 95°C for 3 min, followed by 40 cycles at 95°C for 20 sec, 64°C for 40 sec, and 72°C for 1 min using a melting curve program (increasing the temperature from 55°C to 95°C at 0.5°C per 10 sec) and continuous fluorescence measurements. Sequence-specific products were identified by generating a melting curve in which the C_T_ value represented the cycle number at which a fluorescent signal was significantly greater than background, and relative gene expression was quantified using the 2^–ΔΔCT^ method [31]. For the control, the relative quantification of gene expression was normalized to the C_T_ value of the untreated oviduct.

### In Situ Hybridization Analysis

For hybridization probes, PCR products were generated from cDNA with the primers used for RT-PCR analysis. The products were gel-extracted and cloned into pGEM-T vector (Promega). After verification of the sequences, plasmids containing gene sequences were amplified with T7- and SP6-specific primers (T7∶5′-TGT AAT ACG ACT CAC TAT AGG G-3′; SP6∶5′-CTA TTT AGG TGA CAC TAT AGA AT-3′) then digoxigenin (DIG)-labeled RNA probes were transcribed using a DIG RNA labeling kit (Roche Applied Science, Indianapolis, IN). The signal was visualized by exposure to a solution containing 0.4 mM 5-bromo-4-chloro-3-indolyl phosphate, 0.4 mM nitroblue tetrazolium, and 2 mM levamisole (Sigma).

### Immunohistochemistry

Immunocytochemical localization of AHCYL1 protein in the chicken oviduct was performed as described previously [32] using an anti-human AHCYL1 monoclonal antibody (catalog number: ab56761; abcam, Cambridge, UK) at a final dilution of 1∶500 (1 µg/ml). Antigen retrieval was performed using the boiling citrate method as described previously [32]. Negative controls included substitution of the primary antibody with purified non-immune mouse IgG at the same final concentration.

### Western Blot Analyses

Chicken oviduct cells were isolated and cultured with minor modifications as we described previously [33]. Cells were grown in DMEM-F12 containing 10% charcoal-stripped FBS until they were 80% confluent and starved in serum free medium for 24 h before DES treatment. The protein content was determined using the Bradford protein assay (Bio-Rad, Hercules,CA) with bovine serum albumin (BSA) as the standard. Proteins were denatured, separated using 10% SDS-PAGE and transferred to nitrocellulose. Blots were developed using enhanced chemiluminescence detection (SuperSignal West Pico, Pierce, Rockford, IL) and quantified by measuring the intensity of light emitted from correctly sized bands under ultraviolet light using a ChemiDoc EQ system and Quantity One software (Bio-Rad, Hercules, CA). Immunoreactive AHCYL1 and phosphorylated ERK1/2 protein was detected using an anti-human AHCYL1 monoclonal antibody (catalog number: ab56761; abcam, Cambridge, UK) at a final dilution 1∶500 and anti-mouse phospho-ERK1/2 monoclonal IgG (catalog number: sc-7383; Santa Cruz, CA) at a final dilution 1∶1000, respectively. As a loading control, western blotting with mouse anti-beta actin IgG (catalog number: sc-47778; Santa Cruz, CA) or anti-rabbit total ERK1/2 polyclonal IgG (catalog number: 9102; Cell signaling Technology) was performed.

### Immunofluorescence Microscopy

Oviduct cells obtained from laying hens were examined for AHCYL1 protein expression patterns by immunofluorescence microscopy as described previously [34]. Briefly, oviduct cells were cultured to 80% confluency in charcoal-stripped FBS to remove sex steroids, starved and then treated with 2 µg/ml DES and 10 µM U0126 (ERK1/2 inhibitor) for 24 h. Each type of cell was seeded onto Lab-Tek chamber slide (Nalge Nunc International, Rochester, NY). After 24 h, cells were fixed with −20°C methanol and immunofluorescence staining was performed using an anti-human AHCYL1 monoclonal antibody (catalog number: ab56761; abcam, Cambridge, UK). Cells were then incubated with Alexa Fluor 488 rabbit anti-mouse IgG secondary antibody (A21204, Invitrogen). Slides were overlayed with DAPI before images were captured using a Zeiss confocal microscope LSM710 (Carl Zeiss) fitted with a digital microscope camera AxioCam using Zen 2009 software.

### Target-specific siRNAs for AHCYL1 Knockdown

For messenger RNA sequences of chicken AHCYL1 (NM_001030913.1), three potential small interfering RNA target sites were determined using the Invitrogen design program. The most effective target sequence (GTG AGA AGC AGC AAA CCA A) was screened out and synthesized. Silencer Negative Control 1 siRNA (Ambion, Austin, TX), with no homology to any known gene, was used as a negative control. Down-regulation of AHCYL1 expression was confirmed by quantitative PCR and Western blotting analyses. The information on primers used for q-PCR is described in Table 2.

**Table 2:** Primers used for quantitative RT-PCR.

Gene	Sequence (5′→3′):Forward and Reverse	GenBankAccession No.	Product Size (bp)
*AHCYL1*	GCCATTCCAACACGGAGAT	NM_001030913.1	179
	GATAGAGAGGACAAAGGTGGG		
*CTSB*	CTGGAGAAATGTGAATGGCG	NM_205371.1	157
	CTGGGGACTGAAGACTGGCT		
*CTSC*	GCACTACGGCATCACATCCT	XM_417207.2	151
	AACCTGCTCCCCTGACACAT		
*CTSS*	TGCCACGTGCTCCAAGTATG	NM_001031345.1	173
	CGTGGTTCACCTCCTGTGTG		
*ERRFI1*	AGGAGAGGAGGAGAGTATGG	XP_417525.2	125
	CTGGAACACAGAAGCAGAAC		
*PTN*	CCCTGCTGAACCCAGTGATA	XM_416358.2	174
	AAAATGCCCCCATCCTCTC		
*GAPDH*	CAGAACATCATCCCAGCGTC	NM_204305	133
	GGCAGGTCAGGTCAACAACA		

### Transfection

Chicken oviduct cells were treated with specific AHCYL1 siRNA or controls that included naïve treatment (no siRNA or Lipofectamine 2000) and sham treatment (Lipofectamine 2000 only). Transfection of siRNA was according to the manufacturer’s procedure. To analyze the effect of DES on chicken oviduct cells, DES was added to the culture medium 48 h post-transfection and the incubation continued for either 6 or 24 h. Using red fluorescein-labeled control siRNA duplexes (Invitrogen), we estimated that more than 95% of the cells were transfected.

### MicroRNA Target Validation Assay

The 3′-UTR of *AHCYL1* was cloned and confirmed by sequencing. The 3′-UTR was subcloned between the eGFP gene and the bovine growth hormone (bGH) poly-A tail in pcDNA3eGFP (Clontech, Mountain View, CA) to generate the eGFP-miRNA target 3′-UTR (pcDNA-eGFP-3′UTR) fusion constructs. For the dual fluorescence reporter assay, the fusion constructs containing the *DsRed* gene and either *miR-124a, miR-1602, miR-1612, miR-1669, miR-1710* or *miR-1782* were designed to be co-expressed under control of the CMV promoter (pcDNA-DsRed-miRNA). The pcDNA-eGFP-3′UTR and pcDNA-DsRed-miRNA (4 µg) were co-transfected into 293FT cells using the calcium phosphate method. When the DsRed-miRNA is expressed and binds to the target site of the 3′-UTR downstream of the GFP transcript, green fluorescence intensity decreases due to degradation of the GFP transcript. At 48 h post-transfection, dual fluorescence was detected by fluorescence microscopy and calculated by FACSCalibur flow cytometry (BD Biosciences). For flow cytometry, the cells were fixed in 4% paraformaldehyde and analyzed using FlowJo software (Tree Star Inc., Ashland, OR).

### Calcium Release Assay

Chicken intact or AHCYL1 siRNA knockdowned oviduct cells were treated with various concentrations of DES for 24 h and the supernatant was used to evaluate the release of calcium using Calcium Assay kit (Cayman Chemical, Ann Arbor, MI) according to the manufacturer’s instructions. The 100 µl working detection reagent was added to 10 µl supernatant and gently mixed, incubated at room temperature for 5 min. Calcium concentration was quantified using a microplate reader with a 595 nm absorbance and compared to a calcium standard curve.

### Statistical Analyses

Differences in the variance between untreated and DES-treated oviducts were analyzed using the *F* test, and differences between means were detected using the Student’s *t* test. The probability value of *P*<0.05 was considered statistically significant. Excel (Microsoft, Redmond, WA, USA) was used for statistical analyses.

## Supporting Information

Figure S1 Multiple sequence alignment of chicken, fish and mammalian AHCYL1 proteins. (A) The amino acid sequences of AHCYL1 proteins from chicken (*Gallus gallus)*, human (*Homo sapiens*), orangutan (*Pongo abelii*), mouse (*Mus musculus*), rat (*Rattus norvegicus*), cattle (*Bos taurus*), dog (*Canis lupus familiaris*) and zebrafish (*Danio rerio*) were aligned using Geneious Pro Version 5.04 [27] with default penalties for gap and the protein weight matrix of BLOSUM (Blocks Substitution Matrix). Shaded amino acid sequences are identical among all species examined. Dashes represent gaps among the sequences. The conserved functional domains in AHCYL1 proteins were identified using the Pfam-A family matrix and NCBI conserved domain database.
(TIF)

Figure S2 The phylogenetic tree generated from alignments of primary sequences of chicken, fish and mammalian AHCYL1 proteins. The amino acid sequences were obtained from each GenBank (Table 1). The phylogenetic tree was constructed by the neighbor-joining method using the Geneious program. The numbers next to the branches indicate bootstrap values from 1000 replicates. Bar shows a genetic distance.
(TIF)
