# Supplementary figures and images for: AHCYL1 Is Mediated by Estrogen-Induced ERK1/2 MAPK Cell Signaling and MicroRNA Regulation to Effect Functional Aspects of the Avian Oviduct
Source: PLoS One. 2012 Nov 7;7(11):e49204. doi: 10.1371/journal.pone.0049204 (PMC3492294; doi:10.1371/journal.pone.0049204)

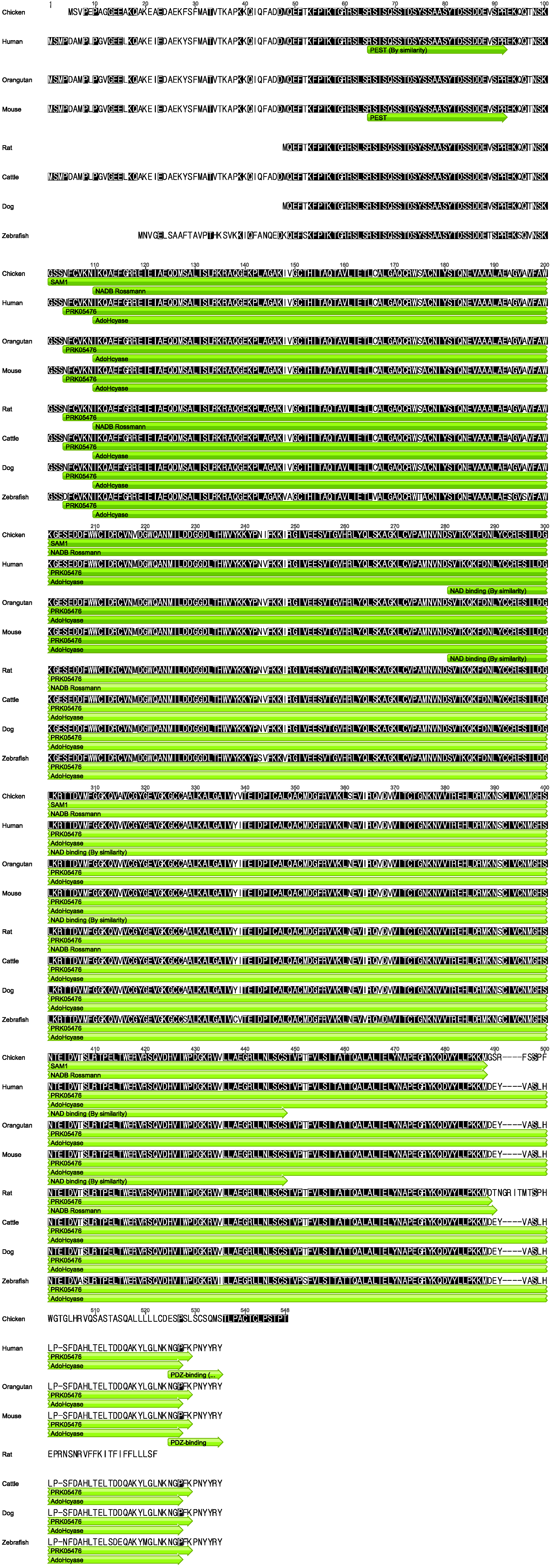

Supplement: Figure S1 — Multiple sequence alignment of chicken, fish and mammalian AHCYL1 proteins. (A) The amino acid sequences of AHCYL1 proteins from chicken (Gallus gallus), human (Homo sapiens), orangutan (Pongo abelii), mouse (Mus musculus), rat (Rattus norvegicus), cattle (Bos taurus), dog (Canis lupus familiaris) and zebrafish (Danio rerio) were aligned using Geneious Pro Version 5.04 [27] with default penalties for gap and the protein weight matrix of BLOSUM (Blocks Substitution Matrix). Shaded amino acid sequences are identical among all species examined. Dashes represent gaps among the sequences. The conserved functional domains in AHCYL1 proteins were identified using the Pfam-A family matrix and NCBI conserved domain database. (TIF) [file pone.0049204.s001.tif]

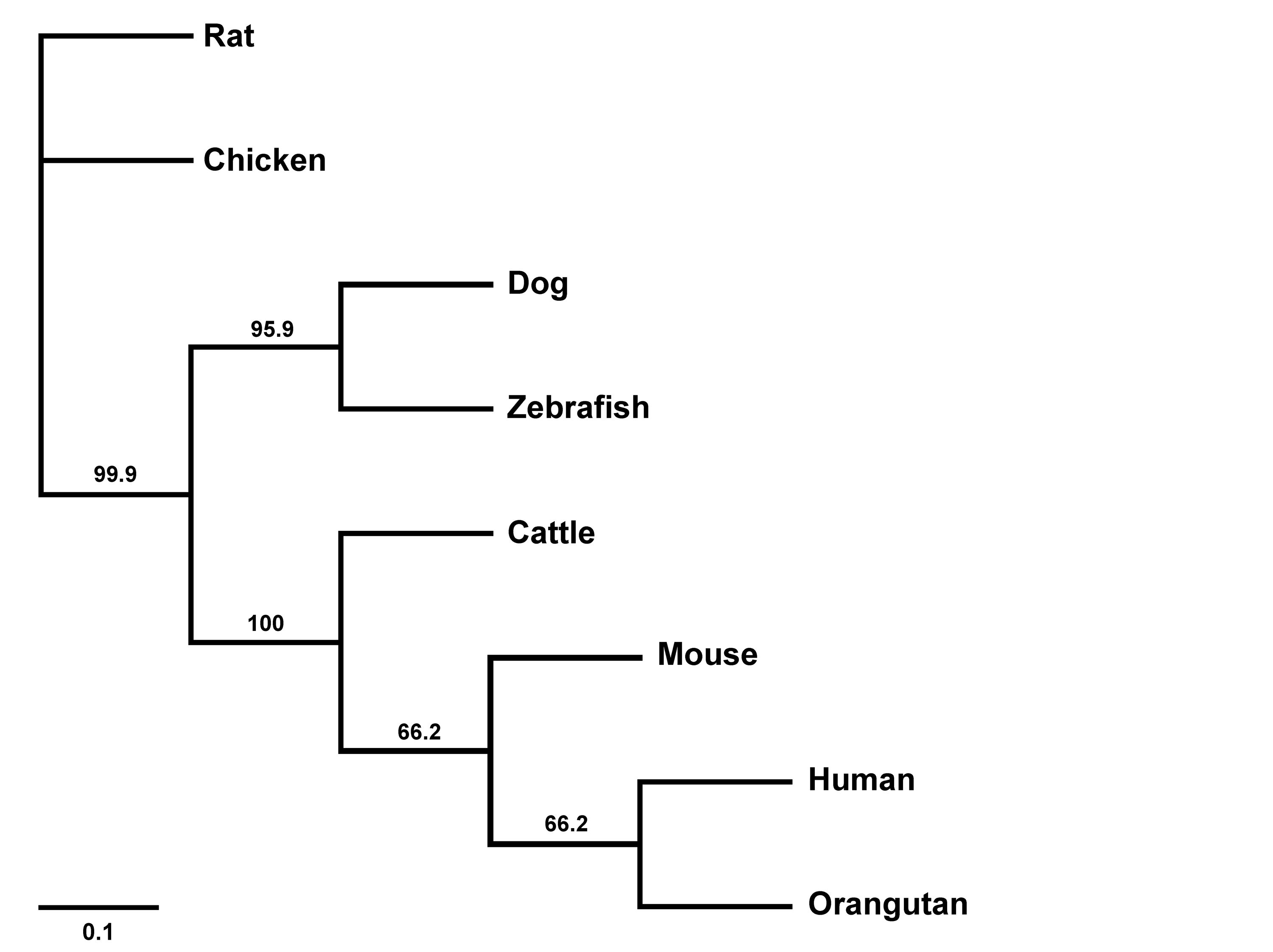

Supplement: Figure S2 — The phylogenetic tree generated from alignments of primary sequences of chicken, fish and mammalian AHCYL1 proteins. The amino acid sequences were obtained from each GenBank (Table 1). The phylogenetic tree was constructed by the neighbor-joining method using the Geneious program. The numbers next to the branches indicate bootstrap values from 1000 replicates. Bar shows a genetic distance. (TIF) [file pone.0049204.s002.tif]
